# Susceptibility and resistance profiles of field and laboratory strains of *Trogoderma granarium* Everts to pirimiphos-methyl, alpha-cypermethrin and spinetoram

**DOI:** 10.7717/peerj.19423

**Published:** 2025-05-13

**Authors:** Muhammad Bukhari, Hafiz Azhar Ali Khan

**Affiliations:** Department of Entomology, University of the Punjab, Lahore, Punjab, Pakistan

**Keywords:** Stored pest management, Insecticide resistance, Khapra beetle

## Abstract

The khapra beetle, *Trogoderma granarium* Everts, 1898, is a significant pest of stored commodities worldwide. Insecticides are heavily relied upon to manage *T. granarium*. However, the long-term usage of insecticides has led to the development of resistance to insecticides, reducing their effectiveness against *T. granarium*. This study investigated variations in susceptibility to pirimiphos-methyl, alpha-cypermethrin and spinetoram in a laboratory and seven field strains of *T. granarium*, using dose-mortality bioassays. Metabolic resistance mechanisms were investigated through synergism studies using piperonyl butoxide (PBO) and *S,S,S*-tributyl phosphorotrithioate (DEF). Susceptibility of the laboratory strain was the highest to all insecticides compared to the field strains. For field strains, the LD_50_ values ranged from 25.9 to 49.6 mg/kg grain for pirimiphos-methyl, 15.0 to 40.5 mg/kg grain for alpha-cypermethrin, and 2.2 to 6.0 mg/kg grain for spinetoram. Compared to the laboratory strain, field strains of *T. granarium* exhibited significant resistance ratios ranging from: 19.9 to 38.1 fold against pirimiphos-methyl, 12.3 to 45.0 fold against alpha-cypermethrin, and 7.3 to 20.0 against spinetoram. In synergism bioassays, there was a significant effect of enzyme inhibitors on enhancing suceptibility to pirimiphos-methyl only in field strains, suggesting P450 monooxygenase and esterases may contribute to pirimiphos-methyl resistance. In conclusion, variable susceptibility to insecticides was observed across different strains of *T. granarium*. Reduced susceptibility to insecticides in field strains compared to the laboratory strain poses challenges for effective control of *T. granarium*.

## Introduction

The khapra beetle, *Trogoderma granarium* Everts, 1898, is a highly destructive pest of stored commodities globally. This species is recognized as both an alien invasive species and a quarantine pest in numerous countries (Gupta et al., 2011; Yadav et al., 2022; Athanassiou, 2023). The infestation and damages caused by *T. granarium* have been reported from the USA, Australia, Canada, Europe (*e.g.*, Greece, Spain and Cyprus), Africa (*e.g.*, Algeria, Libya, Burkina Faso, Morocco, Egypt, Zimbabwe, Sudan, Mauritania, Niger, Senegal and Mali) and Asia (*e.g.*, Afghanistan, Bangladesh, Iran, Saudi Arabia, Myanmar, South Korea, China, Yemen, Sri Lanka, India, Syria, Lebanon, Iraq, Russia, Israel and Pakistan) (French & Venette, 2005; Ahmedani et al., 2007; Honey et al., 2017; Athanassiou, Phillips & Wakil, 2019; Athanassiou, 2023; Qin et al., 2023). *Trogoderma granarium* is primary feeder that causes a huge economic loss to stored commodities by reducing their weight and nutritional quality, mainly by depleting carbohydrate and protein content (French & Venette, 2005; Tripathi, 2018; Kavallieratos, Karagianni & Papanikolaou, 2019; Hassuba et al., 2024). As a result, infested stored commodities face a reduction in their market value (Tushar et al., 2023).

*Trogoderma granarium* is also a major insect pest of stored commodities in Pakistan (Honey et al., 2017; Amjad et al., 2022). Insecticides from different classes are heavily relied on to manage *T. granarium* in Pakistan (Ahmedani et al., 2007; Fiaz et al., 2018; Anwar, Ranjha & Javed, 2020). Insecticides from organophosphate (*e.g.*, pirimiphos-methyl) and pyrethroid (*e.g.*, alpha-cypermethrin) classes are recommended to control stored insect pests, including *T. granarium* (Ali, 2018; Khan, Haider & Khan, 2022). In addition, the regional farmers have reportedly used some non-registered products (*e.g.*, spinosyns) for the management of stored insects, due to their effectiveness against other agricultural pests (Khan et al., 2018; Khan et al., 2023). However, the long-term usage of insecticides often results in reduced susceptibility in insect pests due to the development of resistance to insecticides (Belinato & Martins, 2016; Khan, 2020b; Gong et al., 2023). Several researchers have reported the development of resistance to insecticide in storage pests worldwide (Dyte, 1974; Zettler, 1982; Chaudhry, 1997; Bogamuwa, Weerakoon & Karunaratne, 2002; Boyer, Zhang & Lempérière, 2012; Kang, Pittendrigh & Onstad, 2013; Nayak, Daglish & Phillips, 2015; Collins & Schlipalius, 2018; Attia et al., 2020). Recent reports of resistance to pirimiphos-methyl, permethrin and deltamethrin in a few field strains of *T. granarium* from Pakistan (Feroz et al., 2020; Khan, 2021) are alarming, which necessitates the need to monitor resistance to commonly used insecticides in other geographically isolated strains of *T. granarium*.

Behavioral, penetration, altered target site, and/or metabolic mechanism are typically responsible for reduced susceptibility to insecticides in insect pests (Siddiqui et al., 2023). However, metabolic mechanisms have been found to be the most frequent and challenging resistance mechanism (Karaağaç, 2012; Nauen et al., 2019). The evidence of synergized susceptibility to insecticides when used in binary combinations with enzyme inhibitors, such as piperonyl butoxide (PBO) and *S,S,S*-tributyl phosphorotrithioate (DEF), provides a quick way to assess the presence of metabolic mechanism of resistance in resistant insect species (Ribeiro et al., 2003; Espinosa et al., 2005; Limoee et al., 2007; Paksa, Ladoni & Nasirian, 2012; Yao et al., 2019; Khan, 2020a). Moreover, susceptibility to a particular insecticide also varies with geographical origin of insect species, and also among strains of a specific species collected from different localities (Ribeiro et al., 2003; Wiebe et al., 2017; Agrafioti, Athanassiou & Nayak, 2019; Yao et al., 2019; Anwar, Ranjha & Javed, 2020; Attia et al., 2020; Khan, 2020a; Khan, 2021; Solis-Santoyo et al., 2021; Baliota et al., 2022; Khan, Haider & Khan, 2022; Machuca-Mesa, Turchen & Guedes, 2024).

The recent occurrence of insecticide control failures in Punjab, Pakistan, demands the urgent monitoring of variations in susceptibility to insecticides across different field strains of *T. granarium*. Hence, the present study was planned to assess variations in susceptibility to pirimiphos-methyl, alpha-cypermethrin and spinetoram in different strains of *T. granarium.* These strains were collected from different areas of Punjab, Pakistan. We were also interested in investigating the potential role of metabolic mechanisms in reduced susceptibility to insecticides.

## Materials and Methods

### *Trogoderma granarium* strains

A laboratory and seven field strains of *T. granarium* were used in bioassays. The laboratory strain, which had been collected from stored wheat grains in Lahore (31.5204°N, 74.3587°E) in 2013 and maintained without chemical exposure, acted as the reference strain. Field strains were collected from rice-storage facilities in different cities of Punjab during June to August, 2023: Lahore (31.5204°N, 74.3587°E), Mandi Bahauddin (32.5742°N, 73.4828°E), Rajanpur (29.1044°N, 70.3301°E), Rahim Yar Khan (28.4212°N, 70.2989°E), Narowal (32.1014°N, 74.8800°E), Hafizabad (32.0712°N, 73.6895°E) and Gujranwala (32.1877°N, 74.1945°E). Field strains were coded hereafter as LHR-TG, MBD-TG, RPR-TG, RYK-TG, NWL-TG, HBD-TG and GRW-TG, respectively. All the strains were reared on clean rice grains, *Oryza sativa* L. (var. Basmati-370), at 30 °C, 65% relative humidity, under dark conditions. The strains were reared in the laboratory for at least two generations prior to bioassays.

### Chemicals

Three technical-grade insecticides: spinetoram (a spinosyn; purity>97%), alpha-cypermethrin (a pyrethroid; purity>98%) and pirimiphos-methyl (an organophosphate; purity>99%), two enzyme inhibitors: *S,S,S*-tributyl phosphorotrithioate (DEF; purity>97%) and piperonyl butoxide (PBO; purity = 98%), and acetone (purity = 99.5%) (Chem Service Inc, West Chester, PA, USA) were used in bioassays.

### Grain treatment and dose-mortality bioassays

Grain treatment and dose-mortality bioassays of insecticides were performed on all strains of *T. granarium*, following established protocols (Kavallieratos et al., 2017a; Khan, Haider & Khan, 2022) with minor modifications. Insecticides were dissolved in acetone to prepare solutions of varying concentrations. Six concentrations of each insecticide, resulting in mortalities between 0% and 100% (Robertson et al., 2017) were used in bioassays. The range of concentrations of pirimiphos-methyl and alpha-cypermethrin was 0.25–8 mg kg^−1^ and 04–128 mg kg^−1^ for the Lab-TG strain and field strains, respectively. Spinetoram was bioassayed at 0.1−3.2 mg kg^−1^ and 0.4–12.8 mg kg^−1^ for the Lab-TG strain and field strains, respectively. For the grain treatment, one kg clean rice grains (var. Basmati-370) were sprayed with one ml insecticide solution of a specific concentration *via* AG4-air brush. The grains were rotated manually in a glass jar for 10 min to ensure uniform distribution of the insecticide solution. Grains in the control group were treated with acetone alone. After the grain treatment, 20 g of treated grains were taken in a glass vial (40 ml) and introduced 20 adults (<24 h old) of *T. granarium*. The vials were placed in an incubator set at 30 °C and 65% relative humidity. Mortality data were recorded seven days post-exposure, with adults deemed dead if they did not move when touched with a fine camel-hair brush. All bioassays were repeated three times by preparing fresh insecticide solutions and grain treatment. The entire procedure of bioassays was followed for the Lab-TG and field strains separately.

### Synergism bioassays

Synergism bioassays were performed according to the protocols established by Ribeiro et al. (2003) and Khan (2020c). A solution of either PBO or DEF was prepared in acetone (1 mg ml^−1^). One ml solution of PBO or DEF was applied on the inner-surfaces of a glass vial (20 ml) and left to dry by rotation. After drying, adult beetles were exposed to PBO- or DEF-coated vials for two hours, followed by exposure to varying concentrations of pirimiphos-methyl, alpha-cypermethrin or spinetoram as previously described (dose-mortality bioassays).

### Data analyses

Mortality data from each strain of *T. granarium* in the insecticidal bioassays were used to calculate lethal dose (LD_50_ and LD_99_) values, along with their 95% confidence intervals (CIs), using the natural log probit model *via* PoloPlus software (LeOra-Software, 2005). Mortality corrections were not performed because control group mortality was <3.5%. Any two LD_50_ or LD_99_ values were considered significantly different if their respective 95% CI values did not overlap (Litchfield & Wilcoxon, 1949). Resistance ratios (RRs) were calculated by dividing the LD values of field strains by the corresponding LD value of Lab-TG reference strain. Resistance was categorized as follows: RR <5 (low resistance), RR 5–10 (moderate resistance), and RR >10 (high resistance) (Khan, 2020a). Ratio tests were conducted to compare the LD_50_ and LD_99_ values of field strains with those of the Lab-TG reference strain. Differences were considered significant if the 95% CI of the ratio did not include one (Robertson et al., 2017). The same criterion was applied to determine the significance of LD values for pirimiphos-methyl, alpha-cypermethrin, and spinetoram, with or without PBO or DEF, in the synergism bioassays (Khan & Akram, 2019; Khan, 2020c).

## Results

### Susceptibility of different strains of *Trogoderma granarium* to insecticides

The dose-mortality bioassay results of all insecticides are detailed in Table 1. The Lab-TG strain demonstrated the greatest susceptibility to all tested insecticides. It showed the highest susceptibility towards spinetoram followed by alpha-cypermethrin and pirimiphos-methyl. At the LD_50_ level, alpha-cypermethrin and pirimiphos-methyl were statistically similar, as indicated by overlapping 95% CIs. In the case of pirimiphos-methyl, the LD_50_ and LD_99_ values of field strains ranged from 25.9 to 49.6 mg/kg grain and 570.1 to 2,110.2 mg/kg grain, respectively. At the LD_50_ level, the NWL-TG and MBD-TG strains showed the highest susceptibility to pirimiphos-methyl followed by LHR-TG, RPR-TG, RYK-TG, HBD-TG and GRW-TG strains. For alpha-cypermethrin, the LD_50_ and LD_99_ values of field strains ranged from 15.0 to 40.5 mg/kg grain and 244.2 to 1,046.9 mg/kg grain, respectively. The RPR-TG was the most susceptible strain than the rest of the field strains with the LD_50_ and LD_99_ values 15.0 and 244.2 mg/kg grain, respectively. In the case of spinetoram, the field strains exhibited the highest susceptibility compared to pirimiphos-methyl and alpha-cypermethrin. The values of spinetoram in field strains were estimated from 2.2 to 6.0 mg/kg and 30.5 to 151.0 mg/kg grain at the LD_50_ and LD_99_ level, respectively. The HBD-TG strain was the least susceptible strain to spinetoram among all field strains (Table 1). In all the cases, the control mortality was less than 4%.

The ratio tests for all insecticides at both LD_50_ and LD_99_ levels showed significant differences between the Lab-TG and field strains, based on the criterion 95% CI of the ratio did not include 1 (Table 1). Compared to the Lab-TG strain, field strains of *T. granarium* showed significant resistance, with RR values ranging from: 19.9 to 38.1 fold (at the LD_50_ level) and 43.6 to 162.3 fold (at the LD_99_ level against pirimiphos-methyl; 16.7 to 40.7 fold (at the LD_50_ level) and 12.3 to 95.2 fold (at the LD_99_ level) against alpha-cypermethrin; 7.3 to 20.0 fold (at the LD_50_ level) and 5.9 to 29.0 (at the LD_99_ level) against spinetoram (Table 1).

**Table 1:** Susceptibility of laboratory and field strains of *Trogoderma granarium* to pirimiphosmethyl, alpha-cypermethrin and spinetoram.

**Insecticide**	**Strain**	**LD**_**50**_ ** (95% CI) (mg/kg)**	**LD**_**99**_ **(95% CI) (mg/kg)**	**Fit of probit line**				**LD**_**50**_ ratio (95% CI)^*^	**LD**_**99**_ ratio (95% CI)^*^
				**Intercept**	**Slope (SE)**	*χ* ^**2**^ **(*df* = 4)**	***p***		
Pirimiphos-methyl	Lab-TG	1.3 (1.1–1.5)	13.0 (8.4–19.3)	−0.2 (0.1)	2.3 (0.2)	1.0	0.9	1	1
	LHR-TG	32.0 (23.2–45.5)	570.1 (266.0–2,276.0)	−2.8 (0.3)	1.9 (0.2)	5.7	0.2	24.6 (19.5–32.7)	43.6 (20.1–96.6)
	MBD-TG	26.8 (21.4–31.6)	998.2 (514.5–1,674.6)	−2.1 (0.2)	1.5 (0.1)	1.0	0.9	20.6 (15.9–28.1)	76.8 (29.6–201.0)
	RPR-TG	39.6 (32.8–49.7)	915.6 (507.9–2,162.7)	−2.7 (0.3)	1.7 (0.2)	0.5	0.9	30.5 (23.8–41.2)	70.4 (29.3–170.4)
	RYK-TG	44.2 (35.6–56.8)	1,236.9 (643.3–3,259.2)	−2.6 (0.4)	1.6 (0.3)	2.2	0.7	34.0 (26.2–46.6)	95.1 (37.0-246.5)
	NWL-TG	25.9 (20.8–32.3)	972.3 (502.0–2,599.0)	−2.1 (0.2)	1.5 (0.2)	0.9	0.9	19.9 (13.3–25.7)	74.8 (52.5–90.3)
	HBD-TG	45.0 (36.9–56.2)	851.6 (488.6–1,902.7)	−3.0 (0.3)	1.8 (0.2)	3.2	0.5	34.6 (27.1–46.6)	65.5 (28.2–153.1)
	GRW-TG	49.6 (38.9–66.4)	2,110.2 (952.7–7,200.7)	−2.4 (0.2)	1.5 (0.1)	2.4	0.6	38.1 (28.5–53.7)	162.3 (53.7–494.8)
Alpha-cypermethrin	Lab-TG	0.9 (0.8–1.1)	11.0 (7.1–20.9)	0.1 (0.1)	2.2 (0.2)	1.7	0.8	1	1
	LHR-TG	36.6 (29.6–46.4)	1,046.9 (553.9–2,674.7)	−2.5 (0.2)	1.6 (0.3)	3.2	0.5	40.7 (30.0–53.3)	95.2 (37.3–242.4)
	MBD-TG	29.2 (24.3–35.2)	447.4 (282.3–854.6)	−2.9 (0.3)	2.0 (0.2)	0.8	0.9	32.4 (24.7–41.2)	40.7 (19.0–86.9)
	RPR-TG	15.0 (12.4–18.1)	244.2 (157.8–451.7)	−2.3 (0.2)	1.9 (0.2)	2.5	0.6	16.7 (12.7–21.3)	22.2 (10.6–46.5)
	RYK-TG	40.5 (32.8–51.2)	1,015.3 (549.8–2,490.4)	−2.7 (0.3)	1.7 (0.4)	3.3	0.5	45.0 (33.3–58.9)	92.3 (37.1–229.3)
	NWL-TG	28.7 (24.0–34.6)	424.9 (270.3–799.3)	−2.9 (0.3)	2.0 (0.3)	3.3	0.5	31.9 (24.3–40.5)	38.6 (18.2–82.0)
	HBD-TG	27.6 (23.1–33.2)	406.7 (259.9–760.3)	−2.9 (0.2)	2.0 (0.2)	0.3	0.9	30.7 (23.4–38.9)	37.0 (17.5–78.13)
	GRW-TG	27.0 (21.6–37.1)	949.4 (495.4–2,489.7)	−2.2 (0.2)	1.5 (0.1)	1.1	0.9	30.0 (22.1–39.4)	86.3 (33.3–223.3)
Spinetoram	Lab-TG	0.3 (0.2–0.4)	5.2 (2.6–18.7)	0.9 (0.1)	1.9 (0.2)	4.6	0.3	1	1
	LHR-TG	3.6 (2.7–4.6)	63.8 (30.6–229.2)	−1.0 (0.1)	1.9 (0.3)	4.8	0.3	12.0 (8.8–15.5)	12.3 (5.2–29.3)
	MBD-TG	2.3 (1.7–3.2)	52.7 (24.9–196.3)	−0.6 (0.1)	1.7 (0.2)	4.8	0.3	7.7 (5.7–10.0)	10.1 (4.3–24.6)
	RPR-TG	2.2 (1.9–2.7)	34.6 (22.2–64.1)	−0.7 (0.1)	2.0 (0.4)	2.1	0.7	7.3 (5.5–9.4)	6.7 (3.0–15.0)
	RYK-TG	2.2 (1.7–2.8)	138.5 (63.1–468.9)	−0.4 (0.1)	1.3 (0.1)	1.0	0.9	7.3 (5.1–9.8)	26.6 (8.5–84.9)
	NWL-TG	3.2 (2.2–5.0)	30.5 (14.2–165.7)	−1.2 (0.2)	2.4 (0.3)	8.5	0.1	10.7 (8.0–13.4)	5.9 (2.8–12.6)
	HBD-TG	6.0 (4.8–7.9)	151.0 (76.4–422.8)	−1.3 (0.2)	1.7 (0.2)	2.3	0.6	20.0 (14.2–26.8)	29.0 (10.4–82.5)
	GRW-TG	3.1 (2.1–4.7)	42.0 (18.9–222.8)	−1.0 (0.1)	2.1 (0.2)	7.1	0.1	10.3 (7.7–13.2)	8.1 (3.7–18.1)

**Notes.**

* significant difference between LD_50_ or LD_99_ values of field- and Lab-TG strains based on 95% CI of the ratio did not include one.

### Synergism bioassays of insecticides with PBO or DEF

In the Lab-TG strain, neither PBO nor DEF synergized susceptibility to pirimiphos-methyl, alpha-cypermethrin, or spinetoram (Tables 2–4). A significant effect of PBO and DEF on pirimiphos-methyl susceptibility was observed across all field strains of *T. granarium*. For example, in the case of bioassays of pirimiphos-methyl along with PBO, susceptibility to pirimiphos-methyl at LD_50_ and LD_99_ levels was enhanced by: 1.6 and 2.1 fold, respectively, for the LHR-TG strain; 1.5 and 2.2 fold, respectively, for the MBD-TG strain; 1.8 and 2.8 fold, respectively, for the RPR-TG strain; 2.2 and 2.5 fold, respectively, for the RYK-TG strain; 1.8 and 2.8 fold, respectively, for the NWL-TG strain; 2.7 and 2.6 fold, respectively, for the HBD-TG strain; 3.5 and 14.8 fold, respectively, for the GRW-TG strain. Similarly, susceptibility to pirimiphos-methyl at LD_50_ and LD_99_ levels was enhanced by: 1.8 and 3.1 fold, respectively, for the LHR-TG strain; 1.7 and 3.6 fold, respectively, for the MBD-TG strain; 1.7 and 1.6 fold, respectively, for the RPR-TG strain; 2.3 and 4.2 fold, respectively, for the RYK-TG strain; 2.2 and 4.2 fold, respectively, for the NWL-TG strain; 2.6 and 2.0 fold, respectively, for the HBD-TG strain; 2.5 and 9.6 fold, respectively, for the GRW-TG strain. In addition, the synergism ratios (SR), in most of the cases, were significant in bioassays of pirimiphos-methyl along with PBO or DEF (Table 2). However, the susceptibility to alpha-cypermethrin (Table 3) and spinetoram (Table 4) remained statistically unchanged with the addition of either PBO or DEF in all field strains. In all the cases, the control mortality was less than 4%.

**Table 2:** Effectiveness of pretreatment with either PBO or DEF 2 h before application of pirimiphos-methyl to laboratory and field strains of *Trogoderma granarium*.

**Insecticide**	**Strain**	**LD**_**50**_ ** (95% CI) (mg/kg)**	**LD**_**99**_ ** (95% CI) (mg/kg)**	**Fit of probit line**				Synergism ratio^*^ **at LD**_**50**_ ** (95% CI)**	Synergism ratio^*^ **at LD**_**99**_ **(95% CI)**
				**Intercept**	**Slope (SE)**	*χ* ^**2**^ **(*df* = 4)**	*P*		
Pirimiphos-methyl+PBO	Lab-TG	1.2 (1.0–1.5)	11.6 (7.7–21.0)	−0.2 (0.1)	2.4 (0.2)	2.4	0.7	1.1 (0.8–1.3)	1.1 (0.5–2.0)
Pirimiphos-methyl+DEF	Lab-TG	1.2 (0.9–1.7)	9.5 (5.1–33.4)	−0.3 (0.1)	2.6 (0.3)	4.4	0.4	1.1 (0.8–1.2)	1.4 (0.7–2.3)
Pirimiphos-methyl+PBO	LHR-TG	19.7 (16.4–23.2)	274.3 (166.1–580.7)	−2.6 (0.3)	2.0 (0.2)	0.7	0.9	1.6 (1.2–2.1)	2.1 (0.9–4.9)
Pirimiphos-methyl+DEF	LHR-TG	17.5 (12.8–24.4)	181.4 (92.5–693.1)	−2.8 (0.2)	2.3 (0.3)	3.7	0.4	1.8 (1.4–2.4)	3.1 (1.4–6.8)
Pirimiphos-methyl+PBO	MBD-TG	17.4 (13.9–20.4)	454.9 (234.2–1,307.1)	−2.0 (0.3)	1.6 (0.2)	0.4	0.9	1.5 (1.1–2.1)	2.2 (1.2–4.9)
Pirimiphos-methyl+DEF	MBD-TG	15.9 (11.3–22.3)	275.2 (123.8—1,393.0)	−2.3 (0.4)	1.9 (0.3)	3.1	0.5	1.7 (1.2–2.3)	3.6 (1.3–10.3)
Pirimiphos-methyl+PBO	RPR-TG	21.8 (15.7–32.1)	323.7 (142.7–1,774.5)	−2.7 (0.3)	2.0 (0.4)	3.4	0.5	1.8 (1.4–2.4)	2.8 (1.1–7.4)
Pirimiphos-methyl+DEF	RPR-TG	23.6 (19.0–30.1)	577.7 (289.8–1,730.9)	−2.3 (0.2)	1.7 (0.2)	2.1	0.7	1.7 (1.2–2.3)	1.6 (0.5–4.8)
Pirimiphos-methyl+PBO	RYK-TG	19.8 (15.9–25.0)	497.2 (253.8–1,448.4)	−2.2 (0.3)	1.7 (0.2)	2.1	0.7	2.2 (1.6–3.1)	2.5 (1.3–7.9)
Pirimiphos-methyl+DEF	RYK-TG	19.3 (16.0–23.6)	291.4 (173.6–634.5)	−2.5 (0.3)	2.0 (0.2)	2.4	0.7	2.3 (1.7–3.1)	4.2 (1.5–11.7)
Pirimiphos-methyl+PBO	NWL-TG	14.8 (11.9–18.3)	341.2 (185.5–886.3)	−1.9 (0.3)	1.7 (0.2)	1.3	0.9	1.8 (1.3–2.4)	2.8 (1.4–8.6)
Pirimiphos-methyl+DEF	NWL-TG	11.9 (7.2–18.3)	229.8 (90.0–2.508.6)	−2.0 (0.3)	1.8 (0.3)	4.7	0.3	2.2 (1.6–2.9)	4.2 (1.5–12.0)
Pirimiphos-methyl+PBO	HBD-TG	16.4 (13.3–20.2)	328.3 (184.0–801.5)	−2.2 (0.2)	1.8 (0.4)	2.3	0.7	2.7 (2.0–3.7)	2.6 (1.1-6.9)
Pirimiphos-methyl+DEF	HBD-TG	17.6 (14.2–22.0)	423.1 (222.9–1.161.5)	−2.1 (0.3)	1.7 (0.2)	1.0	0.9	2.6 (1.9–3.5)	2.0 (0.7–5.7)
Pirimiphos-methyl+PBO	GRW-TG	14.1 (9.9–19.9)	142.9 (72.3–598.0)	−2.7 (0.3)	2.3 (0.2)	4.3	0.4	3.5 (2.6–4.8)	14.8 (4.9–44.2)
Pirimiphos-methyl+DEF	GRW-TG	20.1 (14.2–30.0)	219.3 (102.0–1.179.5)	−2.9 (0.3)	2.2 (0.2)	4.5	0.4	2.5 (1.8–3.4)	9.6 (3.1–29.4)

**Notes.**

* synergism ratio calculated by dividing LD_50_ or LD_99_ of pirimiphos-methyl alone with the LD_50_ or LD_99_ of pirimiphos-methyl + PBO or DEF.

**Table 3:** Effectiveness of pretreatment with either PBO or DEF 2 h before application of alphacypermethrin to laboratory and field strains of *Trogoderma granarium*.

**Insecticide**	**Strain**	**LD**_**50**_ ** (95% CI) (mg/kg)**	**LD**_**99**_ ** (95% CI) (mg/kg)**	**Fit of probit line**				Synergism ratio^*^ **at LD**_**50**_ ** (95% CI)**	Synergism ratio^*^ **at LD**_**99**_ **(95% CI)**
				**Intercept**	**Slope (SE)**	*χ* ^**2**^ **(*df* = 4)**	*P*		
Alpha-cypermethrin +PBO	Lab-TG	1.1 (0.7–1.3)	10.2 (6.1–24.3)	−0.1 (0.1)	2.3 (0.2)	5.0	0.3	0.8 (0.7–1.1)	1.1 (0.5–1.9)
Alpha-cypermethrin +DEF	Lab-TG	0.9 (0.6–1.2)	8.2 (5.0–18.0)	0.1 (0.1)	2.4 (0.2)	4.6	0.3	1.0 (0.8–1.3)	1.3 (0.7–2.4)
Alpha-cypermethrin +PBO	LHR-TG	35.2 (27.3–47.0)	2,307 (969.4–8,998.1)	−1.9 (0.2)	1.3 (0.1)	1.6	0.8	1.0 (0.7–1.5)	0.5 (0.1–1.7)
Alpha-cypermethrin +DEF	LHR-TG	36.0 (29.3–45.1)	900.1 (495.5–2,152.9)	−2.6 (0.3)	1.7 (0.2)	0.5	0.9	1.0 (0.7–1.3)	1.2 (0.4–3.3)
Alpha-cypermethrin +PBO	MBD-TG	28.9 (24.0–35.1)	493.6 (305.2–970.4)	−2.7 (0.3)	1.9 (0.2)	3.2	0.5	1.0 (0.5–1.5)	0.9 (0.4–4.0)
Alpha-cypermethrin +DEF	MBD-TG	30.4 (23.5–39.9)	326.9 (185.7–832.2)	−3.3 (0.3)	2.3 (0.3)	4.7	0.3	1.0 (0.7–1.2)	1.4 (0.6–2.8)
Alpha-cypermethrin +PBO	RPR-TG	16.4 (13.9–19.4)	176.7 (122.1–294.0)	−2.7 (0.3)	2.3 (0.1)	0.6	0.9	0.9 (0.7–1.5)	1.4 (0.7–2.7)
Alpha-cypermethrin +DEF	RPR-TG	15.3 (10.6–21.3)	221.3 (111.8–795.4)	−2.4 (0.3)	2.0 (0.2)	6.7	0.2	1.0 (0.8–1.3)	1.1 (0.5–2.3)
Alpha-cypermethrin +PBO	RYK-TG	42.1 (31.4–60.3)	5,116.4 (1,698.8–31,969.0)	−1.8 (0.2)	1.1 (0.1)	2.1	0.7	1.0 (0.6–1.4)	0.2 (0.0–1.0)
Alpha-cypermethrin +DEF	RYK-TG	30.0 (21.6–43.1)	762.6 (326.6–3,590.0)	−2.4 (0.3)	1.7 (0.2)	5.0	0.3	1.4 (0.9–1.8)	1.3 (0.5–3.7)
Alpha-cypermethrin +PBO	NWL-TG	22.6 (18.8–27.2)	349.8 (224.1–651.1)	−2.7 (0.2)	2.0 (0.4)	3.6	0.5	1.3 (0.9–1.6)	1.2 (0.6–2.6)
Alpha-cypermethrin +DEF	NWL-TG	25.7 (15.5–44.8)	469.2 (169.4–6,144.2)	−2.6 (0.2)	1.8 (0.2)	8.4	0.1	1.1 (0.8–1.5)	0.9 (0.4–2.0)
Alpha-cypermethrin +PBO	HBD-TG	24.7 (17.3–35.8)	471.6 (211.0–2,183.0)	−2.5 (0.2)	1.8 (0.1)	6.5	0.2	1.1 (0.8–1.4)	0.87 (0.3–1.9)
Alpha-cypermethrin +DEF	HBD-TG	22.9 (13.1–41.4)	431.6 (149.3–7,719.5)	−2.5 (0.2)	1.8 (0.2)	8.7	0.1	1.2 (0.6–1.6)	0.9 (0.4–2.1)
Alpha-cypermethrin +PBO	GRW-TG	23.1 (19.0–27.9)	432.0 (266.7–854.1)	−2.5 (0.3)	1.8 (0.2)	2.2	0.7	1.2 (0.9–1.6)	2.2 (0.8–5.8)
Alpha-cypermethrin +DEF	GRW-TG	22.4 (18.1–27.7)	629.8 (354.0–1,454.7)	−2.2 (0.2)	1.6 (0.1)	3.6	0.5	1.2 (0.8–1.6)	1.5 (0.5–4.3)

**Notes.**

* synergism ratio calculated by dividing LD_50_ or LD_99_ of alpha-cypermethrin alone with the LD_50_ or LD_99_ of alpha-cypermethrin + PBO or DEF.

**Table 4:** Effectiveness of pretreatment with either PBO or DEF 2 h before application of spinetoram to laboratory and field strains of *Trogoderma granarium*.

**Insecticide**	**Strain**	**LD**_**50**_ ** (95% CI) (mg/kg)**	**LD**_**99**_ ** (95% CI) (mg/kg)**	**Fit of probit line**				Synergism ratio^*^ **at LD**_**50**_ ** (95% CI)**	Synergism ratio^*^ **at LD**_**99**_ **(95% CI)**
				**Intercept**	**Slope (SE)**	*χ* ^**2**^ **(*df* = 4)**	*P*		
Spinetoram+PBO	Lab-TG	0.3 (0.3–0.4)	3.7 (2.4–6.7)	1.1 (0.1)	2.2 (0.2)	1.4	0.9	1.0 (0.7–1.2)	1.4 (0.6–2.7)
Spinetoram+DEF	Lab-TG	0.3 (0.1–0.4)	6.0 (3.5–13.9)	0.9 (0.1)	1.8 (0.3)	1.7	0.9	1.0 (0.7–1.2)	0.9 (0.3–1.8)
Spinetoram+PBO	LHR-TG	2.1 (1.4–3.4)	65.8 (21.7–875.9)	−0.5 (0.1)	1.6 (0.2)	3.2	0.5	1.7 (0.8–2.3)	1.0 (0.3–3.0)
Spinetoram+DEF	LHR-TG	3.4 (2.6–4.8)	117.1 (48.8–514.6)	−0.8 (0.1)	1.5 (0.2)	0.8	0.9	1.1 (0.7–1.5)	0.5 (0.2–1.9)
Spinetoram+PBO	MBD-TG	2.0 (1.6–2.6)	51.9 (26.4–152.1)	−0.5 (0.1)	1.7 (0.2)	2.9	0.6	1.2 (0.9–1.6)	1.0 (0.4–2.9)
Spinetoram+DEF	MBD-TG	1.8 (0.9–4.1)	43.8 (11.2–110.3)	−0.4 (0.1)	1.7 (0.3)	8.6	0.1	1.3 (0.8–1.8)	1.2 (0.4–3.3)
Spinetoram+PBO	RPR-TG	2.3 (1.8–3.0)	80.5 (36.0–306.0)	−0.5 (0.1)	1.5 (0.2)	0.1	1.0	1.0 (0.7–1.3)	0.4 (0.1–1.4)
Spinetoram+DEF	RPR-TG	1.8 (1.0–3.3)	22.0 (8.0–653.2)	−0.5 (0.1)	2.2 (0.2)	8.8	0.1	1.2 (0.9–1.6)	1.6 (0.7–3.4)
Spinetoram+PBO	RYK-TG	1.8 (1.4–2.3)	64.7 (29.8–232.7)	−0.4 (0.1)	1.5 (0.2)	0.2	1.0	1.2 (0.7–1.8)	2.1 (0.5–8.5)
Spinetoram+DEF	RYK-TG	1.7 (1.4–2.1)	39.0 (21.1–102.0)	−0.4 (0.1)	1.7 (0.2)	0.1	1.0	1.3 (0.9–1.7)	2.2 (0.8–12.2)
Spinetoram+PBO	NWL-TG	2.7 (2.2–3.6)	68.7 (33.3–218.8)	−0.7 (0.1)	1.7 (0.2)	2.5	0.6	1.2 (0.8–1.6)	0.4 (0.2-1.2)
Spinetoram+DEF	NWL-TG	2.6 (2.1–3.7)	132.7 (51.0–698.3)	−0.6 (0.1)	1.4 (0.2)	2.0	0.7	1.2 (0.6–1.7)	0.2 (0.1–0.9)
Spinetoram+PBO	HBD-TG	3.4 (2.6–5.0)	108.9 (46.5–455.6)	−0.8 (0.1)	1.5 (0.3)	0.3	1.0	1.8 (0.8–2.6)	1.4 (0.4–5.5)
Spinetoram+DEF	HBD-TG	3.4 (2.9–4.2)	34.1 (20.8–72.5)	−1.3 (0.1)	2.3 (0.3)	2.6	0.6	1.8 (0.6–2.4)	4.4 (0.9–10.4)
Spinetoram+PBO	GRW-TG	2.7 (2.3–3.4)	34.4 (20.5–75.1)	−0.9 (0.1)	2.1 (0.2)	2.6	0.6	1.1 (0.9–1.5)	1.2 (0.5–2.8)
Spinetoram+DEF	GRW-TG	2.8 (1.8–5.1)	39.1 (14.2–614.1)	−0.8 (0.2)	2.0 (0.2)	5.6	0.2	1.1 (0.9–1.4)	1.1 (0.4–2.5)

**Notes.**

* synergism ratio calculated by dividing LD_50_ or LD_99_ of spinetoram alone with the LD_50_ or LD_99_ of spinetoram + PBO or DEF.

## Discussion

For the effective management of stored insect pests, it is crucial to determine susceptibility to insecticides in different laboratory and field strains of a particular pest species (Boyer, Zhang & Lempérière, 2012; Yao et al., 2019). This study investigated the variation in susceptibility to pirimiphos-methyl, alpha-cypermethrin and spinetoram among different field strains of *T. granarium*. The results of the present study revealed differential susceptibility to all the insecticides in different field strains of *T. granarium*. Among all the strains of *T. granarium*, the Lab-TG was the most susceptible one to all the insecticides tested. Overall, field strains exhibited the lowest susceptibility to pirimiphos-methyl and alpha-cypermethrin. In contrast, spinetoram was the most toxic insecticide to all field strains and the laboratory strain of *T. granarium* than pirimiphos-methyl and alpha-cypermethrin. Compared to the Lab-TG strain, field strains exhibited 19.9 to 162.3 fold resistance against pirimiphos-methyl, 12.3 to 95.2 fold resistance against alpha-cypermethrin, and 5.9 to 29.0 resistance against spinetoram.

Organophosphate and pyrethroid insecticides have been in practice to manage stored insect pests in Pakistan for the past four decades (Ali, 2018). Hence, this could be the most probable reason for developing high levels of resistance to pirimiphos-methyl and alpha-cypermethrin in field strains of *T. granarium* in Pakistan. The use of spinetoram against stored insect pests has not yet been recommended by the pest management authorities in Pakistan. However, spinetoram and another spinosyn (spinosad) are recommended against several insect pests of field crops in Pakistan (Ali, 2018). It is assumed that the most probable reasons for showing resistance against spinetoram in *T. granarium* could be due to unintentional exposure to spinetoram at field levels and/or due to the cross-resistance phenomenon (Sparks et al., 2012; Khan, 2020c). Ribeiro et al. (2003) reported that resistance in stored insect species to commonly used insecticides in storage conditions can lead to cross-resistance to insecticides not currently in use. In addition, from the widespread application of organophosphate, pyrethroid, and fumigant insecticides in Pakistan for controlling storage pests may contribute to resistance and cross-resistance to other insecticides. However, it should be confirmed in further studies by selecting resistant strains of *T. granarium* under controlled conditions and conduct analyses of mode of resistance, genetics, and inheritance pattern.

Variations in susceptibility to insecticides is a spatio-temporal phenomenon *i.e.,* it changes with space and time (Chen et al., 2022; Liao et al., 2024). The findings of the present work revealed differential susceptibility to insecticides in different laboratory and field strains of *T. granarium* collected from various localities. These findings align with previous studies showing variable insecticide susceptibility among strains of a particular insect species with different geographical origins. For instance, Attia et al. (2020) reported variable susceptibility to cypermethrin, malathion and pirimiphos-methyl in laboratory and Alexandria strains of *Tribolium castaneum* (Herbst) and *Sitophilus oryzae* (Linnaeus). The LC_50_ values of cypermethrin, malathion and pirimiphos-methyl were 14.3, 1.9 and 0.6 mg/ml, respectively, for the susceptible strain as compared to 32.5, 51.0 and 171.9 mg/ml, respectively, for the Alexandria strain of *T. castaneum*. Similarly, the LC_50_ values of cypermethrin, malathion and pirimiphos-methyl were 0.2, 0.3 and 0.1 mg/ml, respectively, for the susceptible strain as compared to 32.5, 51.0 and 171.9 mg/ml, respectively, for the Alexandria strain of *S. oryzae*. There was a significant difference in susceptibility to spinosad and deltamethrin in different field strains of *Rhyzopertha dominica* (Fabricius) from Taiwan (Chen & Chen, 2013). Variable susceptibility to indoxacarb insecticide has been reported in different Pakistani-field strains of *R. dominica*, *S. oryzae*, *T. castaneum*, *Oryzaephilus surinamensis* (L.) and *S. zeamais* (Motschulsky) (Khan, 2020c). Susceptibility to deltamethrin varied significantly among different laboratory and field strains of *T. granarium* (Feroz et al., 2020). Khan (2021) reported variable susceptibility to pirimiphos-methyl, permethrin, and spinosad in geographically distinct strains of *T. granarium* from Pakistan, which differed from those studied in the present work. There could be different reasons for the variable susceptibility response of different strains of a particular insect species. For instance, geographically distinct strains often exhibit unique histories of biotic and abiotic stresses, varying degree of pesticide exposure, and biological differences. These factors, coupled with variations in their ability to detoxify or metabolize insecticides, can result in differential responses to the same insecticide (Baliota et al., 2022; Chen et al., 2022; Khan, 2023; Liao et al., 2024).

Pirimiphos-methyl, alpha-cypermethrin, and spinetoram have been reported as potential candidates for managing various stored insect pests. For example, Huang & Subramanyam (2005) reported effectiveness of pirimiphos-methyl for controlling *T. castaneum*, *R. dominica*, *Plodia interpunctella* (Hübner) and *Cryptolestes ferrugineus* (Stephens). Rumbos et al. (2018) evaluated capsule suspension and emulsifiable concentrate formulations of pirimiphos-methyl against *R. dominica*, *S. granarius* and *T. confusum*. In that study pirimphos-methyl was the most effective insecticide against *S. granarius* compared to the other tested species. Kavallieratos et al. (2017b) evaluated pirimiphos-methyl, alpha-cypermethrin, or chlorfenapyr-treated polypropylene bags for the control of *R. dominica*, *S. oryzae* and *Prostephanus truncatus* (Horn), and reported satisfactory control of all tested species. In another study, alpha-cypermethrin was proved effective in controlling *Tenebrio molitor* Linnaeus (Athanassiou et al., 2015). Spinetoram is also a potential insecticide for controlling stored insect pests (Vassilakos & Athanassiou, 2023). For instance, Vassilakos et al. (2012) evaluated spinetoram against *R. dominica*, *S. oryzae*, *P. truncatus*, *O. surinamensis*, *T. confusum* and *S. granarius*, and found variable efficacy against the target species. In that study, *R. dominica* and *P. truncatus* were more susceptible species than the rest of the species tested. However, studies also revealed that continuous use of insecticides may develop resistance in insect pests with the passage of time (Collins & Schlipalius, 2018; Attia et al., 2020; Nayak et al., 2020; Khan, Haider & Khan, 2022; Zubair et al., 2022). Varying levels of resistance to pirimiphos-methyl, alpha-cypermethrin and spinetoram in *T. granarium* have been recorded in the present work. Resistance to pirimiphos-methyl and alpha-cypermethrin in *T. granarium* was expected due to the long history of organophosphate and pyrethroid insecticides usage in Pakistan for the management of stored insect pests (Ali, 2018). Although spinetoram is not yet registered for the management of stored insect pests in Pakistan, low levels of resistance to it may result from cross-resistance. Ribeiro et al. (2003) also found cross-resistance to insecticides in field strains of *S. zeamais*.

There are four major mechanisms responsible for the development of insecticide resistance in insect pests: behavioral, penetration, altered target site and metabolic (Siddiqui et al., 2023). Of these, metabolic mechanism is the is the most common resistance mechanism and often presents the greatest challenge (Karaağaç, 2012; Nauen et al., 2019). Insecticides tested in the present study exhibit different modes of action within insect bodies. For example, pirimiphos-methyl is a synaptic insecticide that acts by inhibiting acetylcholinesterase; alpha-cypermethrin is an axonic poison that causes opening of the sodium channels for an extended period; whereas, spinetoram acts by disrupting the GABA-gated chloride channels and the nicotinic acetylcholine receptors in insects (Sparks & Nauen, 2015; Sparks et al., 2021). The evidence of synergized susceptibility to insecticides when used in binary combinations with enzyme inhibitors, such as PBO and DEF, provides a quick way to assess the presence of metabolic mechanism of resistance in resistant insect species (Ribeiro et al., 2003; Espinosa et al., 2005; Limoee et al., 2007; Paksa, Ladoni & Nasirian, 2012; Yao et al., 2019; Khan, 2020a). In the present study, synergism bioassays of pirimiphos-methyl, alpha-cypermethrin or spinetoram in the presence of either PBO or DEF revealed that resistance to pirimiphos-methyl in *T. granarium* could be attributed due to the metabolic mechanism of resistance. Susceptibility to pirimiphos-methyl significantly increased in the presence of PBO or DEF compared to pirimiphos-methyl alone. However, susceptibility to alpha-cypermethrin and spinetoram did not change significantly in the presence of enzyme inhibitors, which suggest the probability of mechanism(s) of resistance other than the metabolic mechanism. For example, the synergists PBO and DEF did not suppress the resistance of *T. granarium* to alpha-cypermethrin. Thus, an altered target-site or knockdown resistance (*kdr*) mechanism was probably involved in resistance to alpha-cypermethrin. Synergized susceptibility to pirimiphos-methyl and non-synergized susceptibility to another pyrethroid and spinosyn (*i.e.,* permethrin and spinosad, respectively) have also been reported in some other strains of *T. granarium* (Khan, 2021). However, some studies have also reported an antagonistic effect of PBO on the susceptibility to pirimiphos-methyl in various insect species (Guedes et al., 1997; Syme et al., 2022). Further *in vitro* studies could provide an insight into the mechanism(s) of insecticide resistance in *T. granarium*.

## Conclusion

In conclusion, the findings of the present study exhibited differential susceptibility and resistance to pirimiphos-methyl, alpha-cypermethrin, and spinetoram in different laboratory and field strains of *T. granarium*. Field strains exhibited relatively higher levels of resistance to pirimiphos-methyl and alpha-cypermethrin compared to spinetoram. Cautious use of insecticides along with integration of non-chemical measures to manage *T. granarium* could delay the development of resistance to insecticide. The high susceptibility of laboratory and field strains of *T. granarium* to spinetoram provides a window to use this product in rotation with others as an option in the integrated management program. However, effective management of insecticide resistance in *T. granarium* requires understanding the genetic basis, mode of inheritance, and resistance mechanisms, which should be addressed in future studies.

##  Supplemental Information

10.7717/peerj.19423/supp-1 Supplemental Information 1 Data of bioassays of different insecticides against *T. granarium* Bioassay data of chlorpyrifos-methyl, alpha-cypermethrin and spinetoram against different laboratory and field strains of *T. granarium*.

## Additional Information and Declarations

### Data Availability

The following information was supplied regarding data availability: The relevant data regarding toxicity of insecticides and synergism bioassays are available in the Supplementary File.
